# 4-Cyano-*N*-ethyl­spiro­[chromene-2,4′-piperidine]-1′-carboxamide

**DOI:** 10.1107/S1600536812051124

**Published:** 2012-12-22

**Authors:** P. Rajalakshmi, N. Srinivasan, R. V. Krishnakumar

**Affiliations:** a Department of Physics, Thiagarajar College, Madurai 625 009, India

## Abstract

The title compound, C_17_H_19_N_3_O_2_, crystallizes with two independent mol­ecules (*A* and *B*) in the asymmetric unit. In both mol­ecules, the pyran ring has a twisted conformation (^5^ *S*_4_), with *Q* = 0.301 (3) Å, θ = 116.7 (6) and ϕ= 213.6 (7)° for mol­ecule *A*, and *Q* = 0.364 (2) Å, θ = 113.7 (3) and ϕ = 213.0 (4)° for mol­ecule *B*. In mol­ecule *B*, the terminal ethyl group is disordered over two orientations with an occupancy ratio of 0.55 (1):0.45 (1). In the crystal, mol­ecules *A* and *B* form very similar but separate *R*_1_^2^(7) motifs through N—H⋯O and C—H⋯O hydrogen bonds. The resulting chains along [001] are inter­linked by weaker C—H⋯O and C—H⋯π inter­actions, forming layers parallel to the *bc* plane.

## Related literature

For related structures, see: Rajalakshmi *et al.* (2012 ▶). For their biological activity, see: Kemnitzer *et al.* (2004 ▶); Mahdavi *et al.* (2011 ▶); Patil *et al.* (2012 ▶); Vosooghi *et al.* (2010 ▶). For puckering parameters, see: Cremer & Pople (1975 ▶). For hydrogen-bond motifs, see: Bernstein *et al.* (1995 ▶).
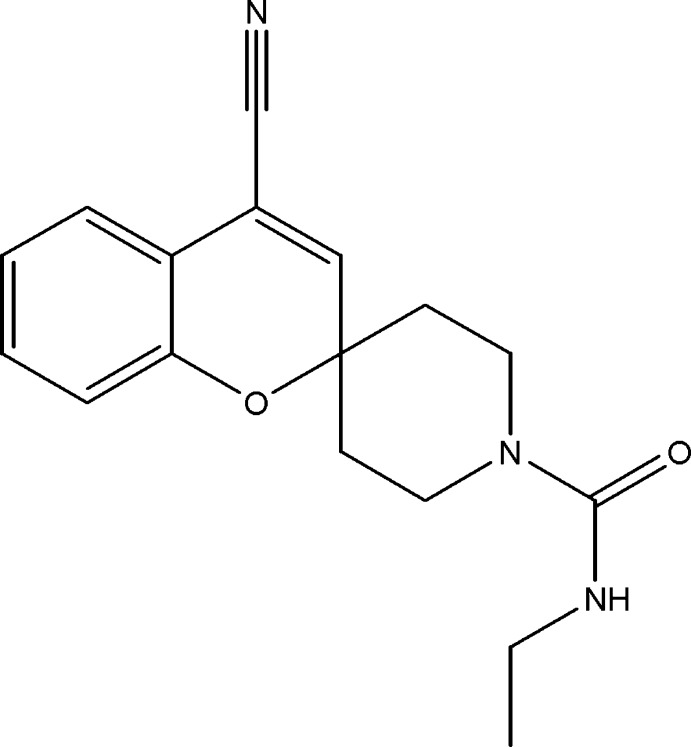


## Experimental

### 

#### Crystal data

C_17_H_19_N_3_O_2_
*M* *_r_* = 297.35
Monoclinic, 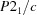

*a* = 22.7845 (8) Å
*b* = 14.3370 (5) Å
*c* = 9.8442 (3) Å
β = 90.783 (1)°
*V* = 3215.42 (19) Å^3^
*Z* = 8
Mo *K*α radiation
μ = 0.08 mm^−1^
*T* = 298 K
0.35 × 0.30 × 0.25 mm

#### Data collection

Bruker Kappa APEXII diffractometer
Absorption correction: multi-scan (*SADABS*; Sheldrick, 2008 ▶) *T*_min_ = 0.972, *T*_max_ = 0.980
26353 measured reflections
5377 independent reflections
3664 reflections with *I* > 2σ(*I*)
*R*_int_ = 0.028

#### Refinement

*R*[*F* ^2^ > 2σ(*F* ^2^)] = 0.050
*wR*(*F* ^2^) = 0.177
*S* = 1.04
5377 reflections
455 parameters
H atoms treated by a mixture of independent and constrained refinement
Δρ_max_ = 0.29 e Å^−3^
Δρ_min_ = −0.20 e Å^−3^

### 

Data collection: *APEX2* (Bruker, 2004 ▶); cell refinement: *SAINT* (Bruker, 2004 ▶); data reduction: *SAINT*; program(s) used to solve structure: *SHELXS97* (Sheldrick, 2008 ▶); program(s) used to refine structure: *SHELXL97* (Sheldrick, 2008 ▶); molecular graphics: *PLUTON* (Spek, 2009 ▶); software used to prepare material for publication: *SHELXL97*.

## Supplementary Material

Crystal structure: contains datablock(s) I, global. DOI: 10.1107/S1600536812051124/ld2085sup1.cif

Structure factors: contains datablock(s) I. DOI: 10.1107/S1600536812051124/ld2085Isup2.hkl

Supplementary material file. DOI: 10.1107/S1600536812051124/ld2085Isup3.cml

Additional supplementary materials:  crystallographic information; 3D view; checkCIF report

## Figures and Tables

**Table 1:** Hydrogen-bond geometry (Å, °) *Cg*1 is the centroid of the C4*B*–C9*B* benzene ring

*D*—H⋯*A*	*D*—H	H⋯*A*	*D*⋯*A*	*D*—H⋯*A*
N3*A*—H1*N*3⋯O2*A* ^i^	0.81 (3)	2.12 (3)	2.915 (3)	170 (3)
N3*B*—H2*N*3⋯O2*B* ^ii^	0.83 (2)	2.19 (3)	2.983 (3)	160 (2)
C11*A*—H11*B*⋯O2*A* ^i^	1.00 (3)	2.27 (3)	3.246 (3)	165 (2)
C11*B*—H11*C*⋯O2*B* ^ii^	0.95 (3)	2.50 (3)	3.344 (3)	147 (2)
C9*B*—H9*B*⋯O2*B* ^iii^	0.92 (3)	2.58 (3)	3.473 (3)	163 (2)
C13*A*—H13*B*⋯*Cg*1^iv^	0.97	2.91	3.843 (3)	163

Symmetry codes: (i) 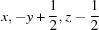; (ii) 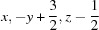; (iii) 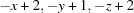; (iv) 
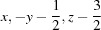
.

**Fig. 1.**
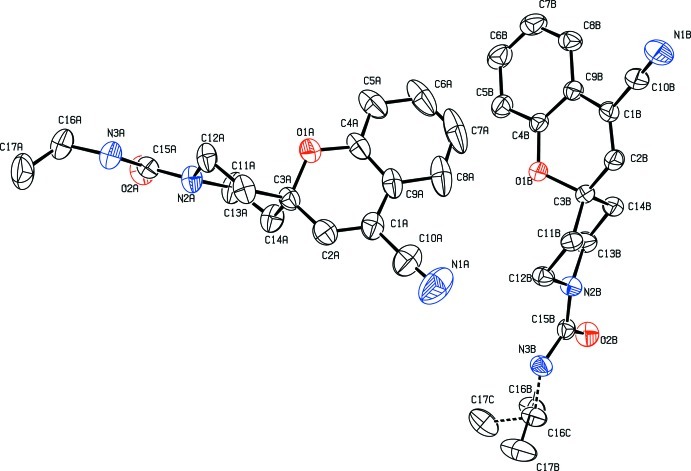
The molecules of the title compound with atom-labeling scheme. Displacement ellipsoids are drawn at 30% probability. For the sake of clarity, H atoms are not shown.

**Fig. 2.**
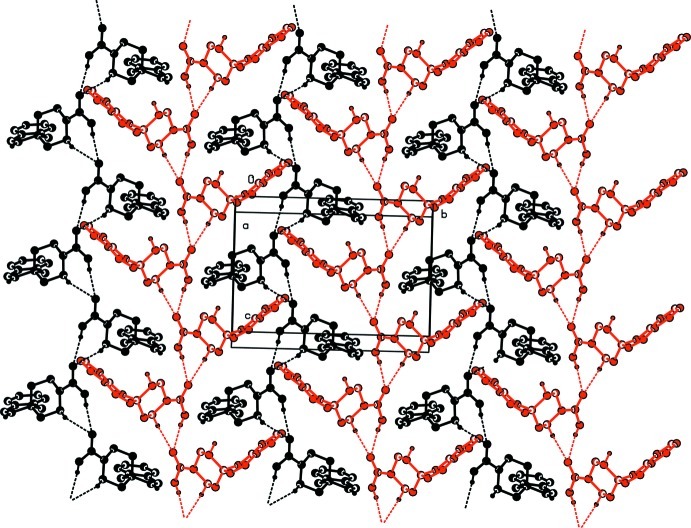
Packing diagram of the title compound, showing formation of the separate R21 (7) motifs by molecules A and B (molecules A are shown in red, molecules B - in black). The cyano and ethyl groups were omitted for clarity as well as the H atoms not involved in the hydrogen bonding.

**Fig. 3.**
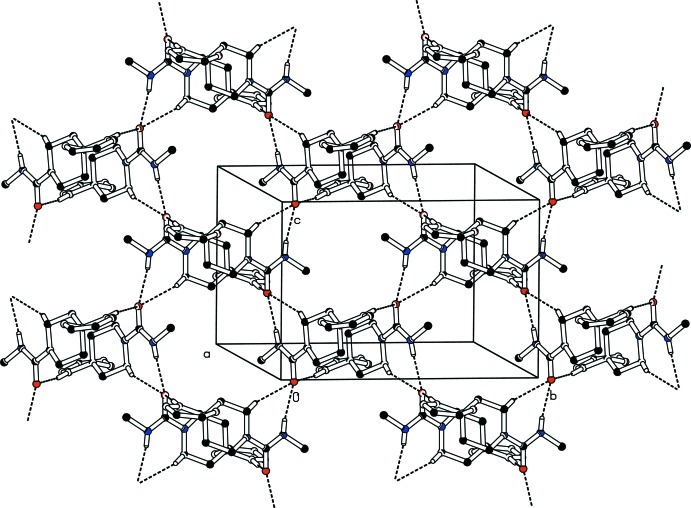
The layers formed by molecules B along (100) plane. Non-essential groups and most hydrogen atoms were omitted for clarity.

## supplementary crystallographic information

### Comment

Chromene with piperidine derivatives are potent agents inducing apoptosis (Kemnitzer *et al.*, 2004). They also exhibit cytotoxic (Vosooghi *et al.*, 2010), antifungal (Mahdavi *et al.*, 2011) and antimycobacterial activities (Patil *et al.*, 2012). In a continuation to our study of the structural features of 1'-benzyl spiro[chromene-2,4'-piperidine]-4-carbonitrile (Rajalakshmi *et al.*, 2012), we report here the crystal structure of the title compound: 4-cyano-*N*-ethylspiro[chromene-2,4'-piperidine]- 1'-carboxamide.

The title compound contains two molecules in the asymmetric unit (Fig. 1). The piperidine ring forms dihedral angles of 11.9 (2)° and 78.2 (1)° for molecule A, 7.9 (8)° and 74.3 (1)° for molecule B, with the *N*-ethyl carboxamide group and chroman ring, respectively. The pyran ring in the molecules A and B (C8/C7/C2/O1/C1/C9) adopts a twisted conformation (^5^S_4_) with O1 and C1 atoms deviating respectively from the mean plane defined by the rest of the atoms by -0.1926 (5) Å and 0.2626 (5) Å in molecule A, and by -0.2375 (4) Å and 0.3063 (5) Å in molecule B. The piperidine ring (C1/C10/C11/N2/C12/C13) adopts a chair conformation (^1^C_4_) with C1 and N2 atoms deviating respectively from the rest of the atoms by -0.6644 (3) Å and 0.6065 (3) Å in molecule A and by -0.6418 (4) Å and 0.6263 (4)Å in molecule B (Cremer & Pople, 1975). In molecule B, the terminal ethyl group is disordered over two positions with refined occupancy ratios of 0.55 (1):0.45 (1).

Molecules A and B form separate chains along [001] through similar *R*^2^_1_(7) motifs (Bernstein *et al.*, 1995) through N—H···O and C—H···O hydrogen bonds. The chains made of molecules B form layers parallel to *bc* plane owing to formation of an additional C9B—H9B···O2B hydrogen bond. The crystal structure also has a noteworthy C—H···π interaction that appears to be a weaker link between molecules A and B resulting in layers parallel to the (100) plane (Fig.2 and Fig.3).

### Experimental

Trimethylsilylcyanide (1.2 mmol) was added to a mixture of *N*-ethyl-4-oxospiro[chroman-2,4'-piperidine]-1'-carboxamide (1.0 mmol) and catalytic amount of ZnI_2_ in dichloromethane (10 vol) under a nitrogen atmosphere. The reaction mixture was stirred at 50°C for 6 h and then cooled down to the room temperature; then diluted HCl (5 ml) was added and stirring continued for additional 2 h. The solution was extracted with ethylacetate (20 ml), dried over Na_2_SO_4_ and evaporated to dryness. The crude product was dissolved in benzene (10 ml), to which tosic acid (0.1 mmol) had been added, and the solution was heated to reflux for 2 h. After completion of the reaction as indicated by TLC, the reaction mixture was concentrated under reduced pressure. The residue was diluted with ethylacetate (20 ml), washed with bicarbonate solution (10 ml) dried and concentrated. The crude product was purified by column chromatography to provide the desired product as colorless solid. Crystals of the title compound were grown from its solution in ethanol by slow evaporation at room temperature.

### Refinement

The positions of the hydrogen atoms bound to N3A, C11A, C13A, N3B, C9B and C11B were allowed to refine with isotropic temperature factors since they participate in the hydrogen-bonding. All other hydrogen atoms were included into the model at geometrically calculated positions (C—H target distance 0.96 Å for methyl hydrogen atoms, 0.93 Å for all others) and refined using a riding model with their *U_iso_* constrained to 1.2 times *U_eq_* (1.5 times for methyl H atoms) of the respective atom to which the hydrogen atom binds. The methyl H atoms involving the C16 atom of molecules A and B were allowed to refine with their torsion angles optimized. In molecule B, the terminal ethyl group C15B and C16B is disordered over two sets of sites the treatment of which converged with occupancy values of 0.55 (1) and 0.45 (1) for the major and minor components, respectively.

### Crystal data

**Table:**

C_17_H_19_N_3_O_2_	*F*(000) = 1264
*M**_r_* = 297.35	*D*_x_ = 1.228 Mg m^−^^3^
Monoclinic, *P*2_1_/*c*	Mo *K*α radiation, λ = 0.71073 Å
Hall symbol: -P 2ybc	Cell parameters from 5377 reflections
*a* = 22.7845 (8) Å	θ = 2–24.5°
*b* = 14.3370 (5) Å	µ = 0.08 mm^−^^1^
*c* = 9.8442 (3) Å	*T* = 298 K
β = 90.783 (1)°	Block, colourless
*V* = 3215.42 (19) Å^3^	0.35 × 0.30 × 0.25 mm
*Z* = 8	

### Data collection

**Table:**

Bruker Kappa APEXII diffractometer	5377 independent reflections
Radiation source: fine-focus sealed tube	3664 reflections with *I* > 2σ(*I*)
Graphite monochromator	*R*_int_ = 0.028
φ and ω scans	θ_max_ = 24.5°, θ_min_ = 1.0°
Absorption correction: multi-scan (*SADABS*; Sheldrick, 2008)	*h* = −26→26
*T*_min_ = 0.972, *T*_max_ = 0.980	*k* = −16→16
26353 measured reflections	*l* = −11→11

### Refinement

**Table:**

Refinement on *F*^2^	Primary atom site location: structure-invariant direct methods
Least-squares matrix: full	Secondary atom site location: difference Fourier map
*R*[*F*^2^ > 2σ(*F*^2^)] = 0.050	Hydrogen site location: inferred from neighbouring sites
*wR*(*F*^2^) = 0.177	H atoms treated by a mixture of independent and constrained refinement
*S* = 1.04	*w* = 1/[σ^2^(*F*_o_^2^) + (0.0985*P*)^2^ + 0.7688*P*] where *P* = (*F*_o_^2^ + 2*F*_c_^2^)/3
5377 reflections	(Δ/σ)_max_ < 0.001
455 parameters	Δρ_max_ = 0.29 e Å^−^^3^
0 restraints	Δρ_min_ = −0.20 e Å^−^^3^

### Special details

**Table:**

Geometry. All esds (except the esd in the dihedral angle between two l.s. planes) are estimated using the full covariance matrix. The cell esds are taken into account individually in the estimation of esds in distances, angles and torsion angles; correlations between esds in cell parameters are only used when they are defined by crystal symmetry. An approximate (isotropic) treatment of cell esds is used for estimating esds involving l.s. planes. Least-squares planes (x,y,z in crystal coordinates) and deviations from them (* indicates atom used to define plane) 21.7749 (0.0093) x - 3.7501 (0.0221) y + 1.2007 (0.0105) z = 19.0074 (0.0242) * 0.0040 (0.0012) C10B * -0.0040 (0.0012) C11B * 0.0040 (0.0012) C12B * -0.0040 (0.0012) C13B 0.6065 (0.0031) N2B -0.6644 (0.0031) C1B Rms deviation of fitted atoms = 0.0040 - 7.4187 (0.0441) x + 12.7666 (0.0128) y + 3.1729 (0.0124) z = 1.9928 (0.0318) Angle to previous plane (with approximate esd) = 59.92 ( 0.15 ) * -0.0108 (0.0016) C10A * 0.0109 (0.0016) C11A * -0.0109 (0.0016) C12A * 0.0108 (0.0016) C13A -0.6263 (0.0038) N2A 0.6418 (0.0039) C1A Rms deviation of fitted atoms = 0.0109 4.5322 (0.0409) x - 8.3873 (0.0214) y + 7.7125 (0.0087) z = 2.7977 (0.0417) Angle to previous plane (with approximate esd) = 70.38 ( 0.11 ) * 0.0378 (0.0016) C7A * 0.0217 (0.0009) C9A * -0.0402 (0.0017) C8A * -0.0193 (0.0008) C2A 0.2626 (0.0051) C1A -0.1926 (0.0048) O1A Rms deviation of fitted atoms = 0.0312 5.5265 (0.0340) x - 2.7276 (0.0239) y + 9.3313 (0.0033) z = 11.8513 (0.0329) Angle to previous plane (with approximate esd) = 24.84 ( 0.15 ) * 0.0573 (0.0014) C7B * 0.0322 (0.0008) C9B * -0.0600 (0.0015) C8B * -0.0295 (0.0007) C2B 0.3063 (0.0047) C1B -0.2375 (0.0041) O1B Rms deviation of fitted atoms = 0.0469 13.1321 (0.0221) x - 10.5040 (0.0116) y - 3.6406 (0.0129) z = 2.2898 (0.0197) Angle to previous plane (with approximate esd) = 86.26 ( 0.11 ) * 0.2282 (0.0021) C10A * -0.2326 (0.0021) C11A * -0.2132 (0.0023) C12A * 0.2074 (0.0023) C13A * 0.2229 (0.0020) N2A * -0.2128 (0.0019) C1A Rms deviation of fitted atoms = 0.2197 16.1311 (0.0326) x - 9.6533 (0.0246) y - 2.1927 (0.0143) z = 5.2730 (0.0209) Angle to previous plane (with approximate esd) = 11.90 ( 0.24 ) * 0.0010 (0.0008) O2A * -0.0020 (0.0016) C14A * 0.0019 (0.0015) N3A * -0.0009 (0.0007) C15A Rms deviation of fitted atoms = 0.0015 13.1321 (0.0221) x - 10.5040 (0.0116) y - 3.6406 (0.0129) z = 2.2898 (0.0197) Angle to previous plane (with approximate esd) = 11.90 ( 0.24 ) * 0.2282 (0.0021) C10A * -0.2326 (0.0021) C11A * -0.2132 (0.0023) C12A * 0.2074 (0.0023) C13A * 0.2229 (0.0020) N2A * -0.2128 (0.0019) C1A Rms deviation of fitted atoms = 0.2197 4.0940 (0.0233) x - 7.7943 (0.0130) y + 8.0459 (0.0062) z = 3.0223 (0.0189) Angle to previous plane (with approximate esd) = 78.16 ( 0.09 ) * -0.0636 (0.0019) C9A * 0.1923 (0.0017) C1A * -0.0711 (0.0019) C8A * 0.0861 (0.0018) C7A * 0.0441 (0.0018) C2A * -0.1878 (0.0017) O1A Rms deviation of fitted atoms = 0.1229 19.4554 (0.0116) x - 7.4511 (0.0119) y + 0.1595 (0.0105) z = 13.7691 (0.0188) Angle to previous plane (with approximate esd) = 62.69 ( 0.09 ) * 0.2327 (0.0017) C10B * -0.2207 (0.0017) C11B * -0.2127 (0.0017) C12B * 0.2240 (0.0017) C13B * 0.2129 (0.0017) N2B * -0.2363 (0.0016) C1B Rms deviation of fitted atoms = 0.2234 19.0116 (0.1135) x - 7.7504 (0.1004) y - 1.1689 (0.0356) z = 12.1762 (0.2227) Angle to previous plane (with approximate esd) = 7.92 ( 0.76 ) * 0.0316 (0.0019) O2B * -0.0581 (0.0036) C14B * 0.0512 (0.0032) N3B * -0.0247 (0.0015) C15B_a Rms deviation of fitted atoms = 0.0436 19.4554 (0.0116) x - 7.4511 (0.0119) y + 0.1595 (0.0105) z = 13.7691 (0.0188) Angle to previous plane (with approximate esd) = 7.92 ( 0.76 ) * 0.2327 (0.0017) C10B * -0.2207 (0.0017) C11B * -0.2127 (0.0017) C12B * 0.2240 (0.0017) C13B * 0.2129 (0.0017) N2B * -0.2363 (0.0016) C1B Rms deviation of fitted atoms = 0.2234 4.1399 (0.0218) x - 2.4193 (0.0136) y + 9.5114 (0.0026) z = 11.0331 (0.0176) Angle to previous plane (with approximate esd) = 74.34 ( 0.08 ) * -0.0681 (0.0018) C9B * 0.2285 (0.0016) C1B * -0.0966 (0.0017) C8B * 0.1149 (0.0015) C7B * 0.0475 (0.0015) C2B * -0.2262 (0.0014) O1B Rms deviation of fitted atoms = 0.1488
Refinement. Refinement of *F*^2^ against ALL reflections. The weighted *R*-factor *wR* and goodness of fit *S* are based on *F*^2^, conventional *R*-factors *R* are based on *F*, with *F* set to zero for negative *F*^2^. The threshold expression of *F*^2^ > σ(*F*^2^) is used only for calculating *R*-factors(gt) *etc*. and is not relevant to the choice of reflections for refinement. *R*-factors based on *F*^2^ are statistically about twice as large as those based on *F*, and *R*- factors based on ALL data will be even larger.

### Fractional atomic coordinates and isotropic or equivalent isotropic displacement parameters (Å^2^)

**Table:**

	*x*	*y*	*z*	*U*_iso_*/*U*_eq_	Occ. (<1)
O1A	0.64156 (8)	0.54792 (13)	0.5566 (2)	0.0827 (6)	
O2A	0.54458 (9)	0.21606 (13)	0.64983 (17)	0.0765 (5)	
N1A	0.86504 (18)	0.6414 (3)	0.5007 (5)	0.1602 (17)	
N2A	0.59436 (10)	0.32206 (14)	0.52456 (19)	0.0636 (6)	
N3A	0.52207 (10)	0.22847 (16)	0.4292 (2)	0.0679 (6)	
C1A	0.67909 (12)	0.47355 (17)	0.5127 (3)	0.0631 (7)	
C2A	0.66403 (15)	0.61628 (18)	0.6402 (3)	0.0770 (8)	
C3A	0.62636 (19)	0.6657 (2)	0.7180 (3)	0.1049 (11)	
H3A	0.5868	0.6498	0.7208	0.126*	
C4A	0.6473 (3)	0.7387 (3)	0.7913 (4)	0.1313 (18)	
C5A	0.7030 (4)	0.7632 (3)	0.7908 (5)	0.150 (2)	
H5A	0.7155	0.8143	0.8418	0.179*	
C6A	0.7447 (2)	0.7118 (2)	0.7122 (4)	0.1209 (16)	
H6A	0.7843	0.7279	0.7130	0.145*	
C7A	0.72431 (15)	0.63792 (17)	0.6358 (3)	0.0767 (9)	
C8A	0.75991 (13)	0.5822 (2)	0.5441 (3)	0.0773 (8)	
C9A	0.73837 (15)	0.5081 (2)	0.4842 (3)	0.0775 (8)	
C10A	0.64728 (14)	0.43474 (18)	0.3889 (3)	0.0733 (8)	
H10A	0.6423	0.4842	0.3225	0.088*	
H10B	0.6714	0.3866	0.3485	0.088*	
C11A	0.58882 (14)	0.3948 (2)	0.4199 (3)	0.0722 (8)	
C12A	0.62276 (14)	0.3556 (2)	0.6501 (3)	0.0826 (9)	
H12A	0.6278	0.3039	0.7127	0.099*	
H12B	0.5978	0.4015	0.6927	0.099*	
C13A	0.68102 (13)	0.3981 (2)	0.6220 (3)	0.0744 (8)	
H13A	0.7077	0.3493	0.5939	0.089*	
H13B	0.6966	0.4250	0.7054	0.089*	
C14A	0.55232 (11)	0.25439 (16)	0.5395 (2)	0.0550 (6)	
C15A	0.47839 (14)	0.1548 (2)	0.4335 (3)	0.0863 (9)	
H15A	0.4797	0.1262	0.5228	0.104*	
H15B	0.4398	0.1823	0.4210	0.104*	
C16A	0.48610 (19)	0.0829 (3)	0.3323 (5)	0.1428 (18)	
H16A	0.4825	0.1098	0.2433	0.214*	
H16B	0.4566	0.0358	0.3432	0.214*	
H16C	0.5243	0.0554	0.3433	0.214*	
C17A	0.81935 (18)	0.6132 (3)	0.5193 (4)	0.1113 (13)	
O1B	0.82241 (6)	0.54557 (10)	0.91701 (17)	0.0585 (4)	
O2B	1.00094 (7)	0.69692 (11)	1.21492 (17)	0.0658 (5)	
N1B	0.78919 (13)	0.18872 (18)	0.8024 (3)	0.1075 (10)	
N2B	0.95277 (8)	0.63343 (12)	1.03499 (18)	0.0512 (5)	
N3B	1.01500 (10)	0.75979 (14)	1.0100 (3)	0.0654 (6)	
C1B	0.87563 (9)	0.48999 (15)	0.9275 (2)	0.0506 (6)	
C2B	0.77116 (9)	0.50472 (16)	0.9577 (2)	0.0509 (6)	
C3B	0.72738 (11)	0.56214 (19)	1.0024 (3)	0.0669 (7)	
H3B	0.7337	0.6260	1.0106	0.080*	
C4B	0.67375 (12)	0.5239 (2)	1.0350 (3)	0.0795 (8)	
H4B	0.6438	0.5626	1.0645	0.095*	
C5B	0.66405 (12)	0.4301 (2)	1.0248 (3)	0.0782 (8)	
H5B	0.6279	0.4052	1.0482	0.094*	
C6B	0.70809 (11)	0.37264 (19)	0.9795 (2)	0.0649 (7)	
H6B	0.7015	0.3088	0.9723	0.078*	
C7B	0.76231 (9)	0.40935 (16)	0.9444 (2)	0.0523 (6)	
C8B	0.81077 (10)	0.35561 (16)	0.8874 (3)	0.0594 (6)	
C9B	0.86373 (11)	0.39305 (17)	0.8769 (3)	0.0617 (7)	
C10B	0.91981 (10)	0.54055 (15)	0.8409 (2)	0.0536 (6)	
H10C	0.9046	0.5451	0.7486	0.064*	
H10D	0.9559	0.5046	0.8387	0.064*	
C11B	0.93324 (12)	0.63759 (17)	0.8938 (2)	0.0545 (6)	
C12B	0.91192 (11)	0.58582 (17)	1.1254 (2)	0.0606 (6)	
H12C	0.8760	0.6218	1.1315	0.073*	
H12D	0.9292	0.5815	1.2157	0.073*	
C13B	0.89774 (10)	0.48906 (16)	1.0738 (2)	0.0583 (6)	
H13C	0.8681	0.4614	1.1309	0.070*	
H13D	0.9327	0.4506	1.0802	0.070*	
C14B	0.98940 (9)	0.69875 (14)	1.0919 (2)	0.0485 (5)	
C15B	1.0460 (8)	0.8346 (14)	1.0830 (13)	0.070 (3)	0.550 (10)
H15C	1.0681	0.8097	1.1597	0.083*	0.550 (10)
H15D	1.0185	0.8808	1.1158	0.083*	0.550 (10)
C16B	1.0868 (4)	0.8769 (6)	0.9786 (7)	0.116 (4)	0.550 (10)
H16D	1.0641	0.9003	0.9031	0.175*	0.550 (10)
H16E	1.1135	0.8300	0.9473	0.175*	0.550 (10)
H16F	1.1086	0.9271	1.0195	0.175*	0.550 (10)
C15C	1.0598 (9)	0.8312 (15)	1.0386 (15)	0.066 (4)	0.450 (10)
H15E	1.0775	0.8203	1.1274	0.079*	0.450 (10)
H15F	1.0905	0.8279	0.9713	0.079*	0.450 (10)
C16C	1.0324 (5)	0.9228 (5)	1.0349 (9)	0.106 (4)	0.450 (10)
H16G	1.0608	0.9694	1.0601	0.159*	0.450 (10)
H16H	1.0005	0.9243	1.0975	0.159*	0.450 (10)
H16I	1.0179	0.9353	0.9448	0.159*	0.450 (10)
C17B	0.79915 (12)	0.2625 (2)	0.8393 (3)	0.0758 (8)	
H9A	0.7620 (14)	0.477 (2)	0.420 (4)	0.101 (11)*	
H1N3	0.5307 (12)	0.2494 (18)	0.356 (3)	0.066 (8)*	
H4A	0.6205 (17)	0.779 (3)	0.852 (4)	0.133 (13)*	
H11A	0.5669 (13)	0.455 (2)	0.456 (3)	0.093 (9)*	
H11B	0.5679 (12)	0.3651 (18)	0.341 (3)	0.081 (8)*	
H2N3	1.0026 (10)	0.7676 (16)	0.932 (3)	0.052 (7)*	
H9B	0.8947 (12)	0.3614 (18)	0.840 (3)	0.072 (8)*	
H11C	0.9634 (12)	0.6634 (17)	0.839 (3)	0.069 (7)*	
H11D	0.8987 (11)	0.6814 (17)	0.889 (2)	0.061 (7)*	

### Atomic displacement parameters (Å^2^)

**Table:**

	*U*^11^	*U*^22^	*U*^33^	*U*^12^	*U*^13^	*U*^23^
O1A	0.0796 (13)	0.0716 (12)	0.0965 (15)	0.0050 (9)	−0.0153 (10)	−0.0284 (10)
O2A	0.1061 (15)	0.0809 (12)	0.0426 (10)	−0.0100 (10)	0.0012 (9)	0.0106 (8)
N1A	0.112 (3)	0.164 (4)	0.204 (4)	−0.044 (3)	−0.022 (3)	0.075 (3)
N2A	0.0885 (15)	0.0635 (12)	0.0384 (10)	−0.0106 (11)	−0.0159 (10)	0.0057 (9)
N3A	0.0779 (15)	0.0810 (15)	0.0448 (13)	−0.0190 (12)	−0.0040 (11)	0.0060 (11)
C1A	0.0778 (17)	0.0551 (14)	0.0563 (15)	0.0037 (12)	−0.0063 (13)	−0.0072 (11)
C2A	0.118 (3)	0.0527 (15)	0.0595 (16)	0.0125 (16)	−0.0162 (16)	−0.0056 (12)
C3A	0.152 (3)	0.076 (2)	0.087 (2)	0.030 (2)	−0.013 (2)	−0.0171 (18)
C4A	0.235 (6)	0.072 (3)	0.086 (3)	0.027 (3)	−0.034 (3)	−0.025 (2)
C5A	0.295 (8)	0.065 (2)	0.087 (3)	−0.026 (4)	−0.059 (4)	−0.017 (2)
C6A	0.205 (4)	0.071 (2)	0.086 (2)	−0.043 (3)	−0.057 (3)	0.0088 (19)
C7A	0.120 (3)	0.0477 (15)	0.0611 (17)	−0.0121 (15)	−0.0343 (17)	0.0111 (12)
C8A	0.091 (2)	0.0588 (16)	0.081 (2)	−0.0131 (15)	−0.0197 (16)	0.0239 (15)
C9A	0.092 (2)	0.0634 (18)	0.077 (2)	−0.0001 (16)	0.0027 (17)	0.0111 (15)
C10A	0.112 (2)	0.0593 (15)	0.0487 (15)	−0.0092 (15)	−0.0067 (14)	0.0058 (11)
C11A	0.107 (2)	0.0584 (16)	0.0508 (15)	−0.0139 (16)	−0.0289 (15)	0.0066 (12)
C12A	0.099 (2)	0.104 (2)	0.0443 (14)	−0.0282 (17)	−0.0189 (14)	0.0074 (14)
C13A	0.0867 (19)	0.0840 (18)	0.0522 (15)	−0.0136 (15)	−0.0161 (13)	0.0115 (13)
C14A	0.0685 (15)	0.0546 (13)	0.0419 (13)	0.0066 (11)	0.0011 (11)	0.0011 (10)
C15A	0.083 (2)	0.103 (2)	0.0737 (19)	−0.0332 (17)	−0.0007 (15)	0.0026 (17)
C16A	0.120 (3)	0.124 (3)	0.186 (5)	−0.062 (3)	0.051 (3)	−0.057 (3)
C17A	0.102 (3)	0.103 (3)	0.128 (3)	−0.022 (2)	−0.021 (2)	0.048 (2)
O1B	0.0451 (9)	0.0468 (9)	0.0835 (12)	0.0008 (7)	−0.0023 (8)	0.0072 (8)
O2B	0.0766 (12)	0.0687 (11)	0.0517 (11)	0.0012 (8)	−0.0092 (8)	−0.0092 (8)
N1B	0.109 (2)	0.0665 (17)	0.146 (3)	−0.0219 (15)	−0.0115 (18)	−0.0188 (16)
N2B	0.0563 (11)	0.0536 (11)	0.0437 (10)	−0.0097 (8)	0.0004 (9)	−0.0001 (8)
N3B	0.0703 (14)	0.0572 (13)	0.0681 (15)	−0.0167 (10)	−0.0219 (12)	0.0083 (11)
C1B	0.0402 (11)	0.0490 (12)	0.0626 (15)	−0.0002 (9)	−0.0025 (10)	0.0007 (10)
C2B	0.0428 (12)	0.0586 (14)	0.0512 (13)	−0.0002 (10)	−0.0053 (10)	0.0092 (10)
C3B	0.0570 (15)	0.0717 (16)	0.0721 (17)	0.0086 (12)	0.0044 (13)	0.0073 (13)
C4B	0.0570 (17)	0.103 (2)	0.079 (2)	0.0101 (15)	0.0143 (14)	0.0103 (17)
C5B	0.0547 (16)	0.110 (2)	0.0702 (18)	−0.0109 (16)	0.0091 (14)	0.0177 (16)
C6B	0.0627 (16)	0.0757 (17)	0.0560 (15)	−0.0176 (13)	−0.0053 (12)	0.0149 (12)
C7B	0.0498 (13)	0.0591 (14)	0.0476 (13)	−0.0053 (11)	−0.0102 (10)	0.0085 (10)
C8B	0.0592 (15)	0.0477 (13)	0.0709 (16)	−0.0048 (11)	−0.0127 (12)	0.0031 (11)
C9B	0.0483 (14)	0.0514 (14)	0.0853 (19)	0.0042 (11)	−0.0031 (13)	−0.0038 (12)
C10B	0.0497 (13)	0.0592 (14)	0.0517 (13)	−0.0053 (10)	−0.0033 (10)	−0.0056 (10)
C11B	0.0581 (15)	0.0580 (15)	0.0473 (14)	−0.0105 (12)	−0.0012 (12)	0.0026 (10)
C12B	0.0589 (14)	0.0733 (16)	0.0498 (14)	−0.0075 (12)	0.0068 (11)	0.0032 (11)
C13B	0.0517 (13)	0.0593 (14)	0.0640 (16)	−0.0062 (11)	0.0015 (11)	0.0127 (11)
C14B	0.0501 (12)	0.0438 (12)	0.0517 (14)	0.0066 (10)	−0.0043 (10)	−0.0042 (10)
C15B	0.075 (8)	0.071 (4)	0.063 (7)	−0.024 (5)	−0.001 (5)	0.005 (6)
C16B	0.129 (7)	0.128 (6)	0.092 (5)	−0.075 (6)	−0.017 (5)	0.023 (4)
C15C	0.069 (9)	0.070 (6)	0.058 (9)	−0.028 (6)	−0.003 (6)	0.011 (7)
C16C	0.142 (9)	0.063 (5)	0.114 (7)	−0.022 (5)	0.008 (6)	−0.008 (4)
C17B	0.0683 (17)	0.0597 (17)	0.099 (2)	−0.0108 (13)	−0.0089 (15)	−0.0035 (15)

### Geometric parameters (Å, º)

**Table:**

O1A—C2A	1.374 (3)	N2B—C14B	1.369 (3)
O1A—C1A	1.437 (3)	N2B—C11B	1.455 (3)
O2A—C14A	1.232 (3)	N2B—C12B	1.465 (3)
N1A—C17A	1.134 (4)	N3B—C14B	1.330 (3)
N2A—C14A	1.373 (3)	N3B—C15B	1.466 (19)
N2A—C12A	1.468 (3)	N3B—C15C	1.47 (2)
N2A—C11A	1.470 (3)	N3B—H2N3	0.83 (2)
N3A—C14A	1.331 (3)	C1B—C9B	1.500 (3)
N3A—C15A	1.452 (4)	C1B—C10B	1.513 (3)
N3A—H1N3	0.81 (3)	C1B—C13B	1.519 (3)
C1A—C9A	1.469 (4)	C2B—C3B	1.371 (3)
C1A—C10A	1.516 (3)	C2B—C7B	1.388 (3)
C1A—C13A	1.526 (4)	C3B—C4B	1.381 (4)
C2A—C3A	1.358 (4)	C3B—H3B	0.9300
C2A—C7A	1.409 (4)	C4B—C5B	1.365 (4)
C3A—C4A	1.354 (6)	C4B—H4B	0.9300
C3A—H3A	0.9300	C5B—C6B	1.378 (4)
C4A—C5A	1.318 (8)	C5B—H5B	0.9300
C4A—H4A	1.03 (4)	C6B—C7B	1.391 (3)
C5A—C6A	1.437 (8)	C6B—H6B	0.9300
C5A—H5A	0.9300	C7B—C8B	1.464 (3)
C6A—C7A	1.377 (4)	C8B—C9B	1.326 (3)
C6A—H6A	0.9300	C8B—C17B	1.440 (4)
C7A—C8A	1.459 (4)	C9B—H9B	0.92 (3)
C8A—C9A	1.307 (4)	C10B—C11B	1.515 (3)
C8A—C17A	1.449 (5)	C10B—H10C	0.9700
C9A—H9A	0.94 (3)	C10B—H10D	0.9700
C10A—C11A	1.486 (4)	C11B—H11C	0.95 (3)
C10A—H10A	0.9700	C11B—H11D	1.01 (2)
C10A—H10B	0.9700	C12B—C13B	1.511 (3)
C11A—H11A	1.06 (3)	C12B—H12C	0.9700
C11A—H11B	1.00 (3)	C12B—H12D	0.9700
C12A—C13A	1.490 (4)	C13B—H13C	0.9700
C12A—H12A	0.9700	C13B—H13D	0.9700
C12A—H12B	0.9700	C15B—C16B	1.522 (14)
C13A—H13A	0.9700	C15B—H15C	0.9700
C13A—H13B	0.9700	C15B—H15D	0.9700
C15A—C16A	1.446 (5)	C16B—H16D	0.9600
C15A—H15A	0.9700	C16B—H16E	0.9600
C15A—H15B	0.9700	C16B—H16F	0.9600
C16A—H16A	0.9600	C15C—C16C	1.46 (2)
C16A—H16B	0.9600	C15C—H15E	0.9700
C16A—H16C	0.9600	C15C—H15F	0.9700
O1B—C2B	1.371 (3)	C16C—H16G	0.9600
O1B—C1B	1.454 (2)	C16C—H16H	0.9600
O2B—C14B	1.236 (3)	C16C—H16I	0.9600
N1B—C17B	1.140 (3)		
			
C2A—O1A—C1A	119.4 (2)	C14B—N3B—C15B	113.3 (5)
C14A—N2A—C12A	116.2 (2)	C14B—N3B—C15C	130.6 (6)
C14A—N2A—C11A	121.5 (2)	C14B—N3B—H2N3	120.6 (16)
C12A—N2A—C11A	113.0 (2)	C15B—N3B—H2N3	121.0 (17)
C14A—N3A—C15A	121.8 (2)	C15C—N3B—H2N3	108.1 (18)
C14A—N3A—H1N3	120 (2)	O1B—C1B—C9B	109.72 (17)
C15A—N3A—H1N3	118.2 (19)	O1B—C1B—C10B	104.95 (17)
O1A—C1A—C9A	111.1 (2)	C9B—C1B—C10B	112.0 (2)
O1A—C1A—C10A	103.6 (2)	O1B—C1B—C13B	109.74 (18)
C9A—C1A—C10A	113.6 (2)	C9B—C1B—C13B	111.29 (19)
O1A—C1A—C13A	109.0 (2)	C10B—C1B—C13B	108.90 (17)
C9A—C1A—C13A	110.8 (2)	C3B—C2B—O1B	117.6 (2)
C10A—C1A—C13A	108.4 (2)	C3B—C2B—C7B	121.1 (2)
C3A—C2A—O1A	118.5 (3)	O1B—C2B—C7B	121.1 (2)
C3A—C2A—C7A	121.8 (3)	C2B—C3B—C4B	119.1 (3)
O1A—C2A—C7A	119.6 (3)	C2B—C3B—H3B	120.5
C4A—C3A—C2A	118.9 (5)	C4B—C3B—H3B	120.5
C4A—C3A—H3A	120.6	C5B—C4B—C3B	121.1 (3)
C2A—C3A—H3A	120.6	C5B—C4B—H4B	119.5
C5A—C4A—C3A	122.5 (5)	C3B—C4B—H4B	119.5
C5A—C4A—H4A	116 (2)	C4B—C5B—C6B	119.7 (3)
C3A—C4A—H4A	122 (3)	C4B—C5B—H5B	120.2
C4A—C5A—C6A	120.6 (4)	C6B—C5B—H5B	120.2
C4A—C5A—H5A	119.7	C5B—C6B—C7B	120.5 (3)
C6A—C5A—H5A	119.7	C5B—C6B—H6B	119.8
C7A—C6A—C5A	117.9 (4)	C7B—C6B—H6B	119.8
C7A—C6A—H6A	121.0	C2B—C7B—C6B	118.5 (2)
C5A—C6A—H6A	121.0	C2B—C7B—C8B	116.42 (19)
C6A—C7A—C2A	118.3 (4)	C6B—C7B—C8B	125.0 (2)
C6A—C7A—C8A	124.9 (4)	C9B—C8B—C17B	120.9 (2)
C2A—C7A—C8A	116.7 (2)	C9B—C8B—C7B	120.6 (2)
C9A—C8A—C17A	121.3 (4)	C17B—C8B—C7B	118.5 (2)
C9A—C8A—C7A	121.0 (3)	C8B—C9B—C1B	120.6 (2)
C17A—C8A—C7A	117.7 (3)	C8B—C9B—H9B	122.4 (16)
C8A—C9A—C1A	121.9 (3)	C1B—C9B—H9B	117.0 (16)
C8A—C9A—H9A	118 (2)	C1B—C10B—C11B	112.3 (2)
C1A—C9A—H9A	120 (2)	C1B—C10B—H10C	109.1
C11A—C10A—C1A	113.4 (2)	C11B—C10B—H10C	109.1
C11A—C10A—H10A	108.9	C1B—C10B—H10D	109.1
C1A—C10A—H10A	108.9	C11B—C10B—H10D	109.1
C11A—C10A—H10B	108.9	H10C—C10B—H10D	107.9
C1A—C10A—H10B	108.9	N2B—C11B—C10B	110.37 (19)
H10A—C10A—H10B	107.7	N2B—C11B—H11C	110.2 (15)
N2A—C11A—C10A	110.4 (2)	C10B—C11B—H11C	107.7 (15)
N2A—C11A—H11A	112.2 (16)	N2B—C11B—H11D	107.3 (13)
C10A—C11A—H11A	100.4 (16)	C10B—C11B—H11D	113.7 (13)
N2A—C11A—H11B	106.1 (15)	H11C—C11B—H11D	107 (2)
C10A—C11A—H11B	115.1 (15)	N2B—C12B—C13B	110.99 (19)
H11A—C11A—H11B	113 (2)	N2B—C12B—H12C	109.4
N2A—C12A—C13A	111.2 (2)	C13B—C12B—H12C	109.4
N2A—C12A—H12A	109.4	N2B—C12B—H12D	109.4
C13A—C12A—H12A	109.4	C13B—C12B—H12D	109.4
N2A—C12A—H12B	109.4	H12C—C12B—H12D	108.0
C13A—C12A—H12B	109.4	C12B—C13B—C1B	112.18 (19)
H12A—C12A—H12B	108.0	C12B—C13B—H13C	109.2
C12A—C13A—C1A	113.8 (2)	C1B—C13B—H13C	109.2
C12A—C13A—H13A	108.8	C12B—C13B—H13D	109.2
C1A—C13A—H13A	108.8	C1B—C13B—H13D	109.2
C12A—C13A—H13B	108.8	H13C—C13B—H13D	107.9
C1A—C13A—H13B	108.8	O2B—C14B—N3B	121.2 (2)
H13A—C13A—H13B	107.7	O2B—C14B—N2B	120.5 (2)
O2A—C14A—N3A	121.0 (2)	N3B—C14B—N2B	118.1 (2)
O2A—C14A—N2A	121.2 (2)	N3B—C15B—C16B	104.8 (8)
N3A—C14A—N2A	117.7 (2)	N3B—C15B—H15C	110.8
C16A—C15A—N3A	114.1 (3)	C16B—C15B—H15C	110.8
C16A—C15A—H15A	108.7	N3B—C15B—H15D	110.8
N3A—C15A—H15A	108.7	C16B—C15B—H15D	110.8
C16A—C15A—H15B	108.7	H15C—C15B—H15D	108.9
N3A—C15A—H15B	108.7	C16C—C15C—N3B	109.1 (14)
H15A—C15A—H15B	107.6	C16C—C15C—H15E	109.9
C15A—C16A—H16A	109.5	N3B—C15C—H15E	109.9
C15A—C16A—H16B	109.5	C16C—C15C—H15F	109.9
H16A—C16A—H16B	109.5	N3B—C15C—H15F	109.9
C15A—C16A—H16C	109.5	H15E—C15C—H15F	108.3
H16A—C16A—H16C	109.5	C15C—C16C—H16G	109.5
H16B—C16A—H16C	109.5	C15C—C16C—H16H	109.5
N1A—C17A—C8A	177.0 (5)	H16G—C16C—H16H	109.5
C2B—O1B—C1B	117.26 (16)	C15C—C16C—H16I	109.5
C14B—N2B—C11B	122.66 (18)	H16G—C16C—H16I	109.5
C14B—N2B—C12B	117.30 (18)	H16H—C16C—H16I	109.5
C11B—N2B—C12B	114.26 (18)	N1B—C17B—C8B	178.9 (4)
			
C2A—O1A—C1A—C9A	−38.2 (3)	C1B—O1B—C2B—C3B	153.4 (2)
C2A—O1A—C1A—C10A	−160.6 (2)	C1B—O1B—C2B—C7B	−31.4 (3)
C2A—O1A—C1A—C13A	84.2 (3)	O1B—C2B—C3B—C4B	175.5 (2)
C1A—O1A—C2A—C3A	−157.3 (3)	C7B—C2B—C3B—C4B	0.3 (4)
C1A—O1A—C2A—C7A	27.4 (4)	C2B—C3B—C4B—C5B	0.7 (4)
O1A—C2A—C3A—C4A	−174.1 (3)	C3B—C4B—C5B—C6B	−0.8 (4)
C7A—C2A—C3A—C4A	1.1 (5)	C4B—C5B—C6B—C7B	0.1 (4)
C2A—C3A—C4A—C5A	−0.5 (6)	C3B—C2B—C7B—C6B	−1.0 (3)
C3A—C4A—C5A—C6A	−0.8 (7)	O1B—C2B—C7B—C6B	−176.04 (19)
C4A—C5A—C6A—C7A	1.4 (6)	C3B—C2B—C7B—C8B	176.1 (2)
C5A—C6A—C7A—C2A	−0.8 (4)	O1B—C2B—C7B—C8B	1.1 (3)
C5A—C6A—C7A—C8A	176.1 (3)	C5B—C6B—C7B—C2B	0.8 (3)
C3A—C2A—C7A—C6A	−0.4 (4)	C5B—C6B—C7B—C8B	−176.0 (2)
O1A—C2A—C7A—C6A	174.7 (2)	C2B—C7B—C8B—C9B	13.7 (3)
C3A—C2A—C7A—C8A	−177.5 (3)	C6B—C7B—C8B—C9B	−169.4 (2)
O1A—C2A—C7A—C8A	−2.4 (4)	C2B—C7B—C8B—C17B	−164.4 (2)
C6A—C7A—C8A—C9A	173.9 (3)	C6B—C7B—C8B—C17B	12.5 (4)
C2A—C7A—C8A—C9A	−9.2 (4)	C17B—C8B—C9B—C1B	−179.8 (2)
C6A—C7A—C8A—C17A	−7.6 (4)	C7B—C8B—C9B—C1B	2.1 (4)
C2A—C7A—C8A—C17A	169.3 (2)	O1B—C1B—C9B—C8B	−29.7 (3)
C17A—C8A—C9A—C1A	177.4 (3)	C10B—C1B—C9B—C8B	−145.8 (2)
C7A—C8A—C9A—C1A	−4.2 (4)	C13B—C1B—C9B—C8B	92.0 (3)
O1A—C1A—C9A—C8A	26.8 (4)	O1B—C1B—C10B—C11B	62.5 (2)
C10A—C1A—C9A—C8A	143.1 (3)	C9B—C1B—C10B—C11B	−178.47 (19)
C13A—C1A—C9A—C8A	−94.6 (3)	C13B—C1B—C10B—C11B	−54.9 (2)
O1A—C1A—C10A—C11A	−62.8 (3)	C14B—N2B—C11B—C10B	152.5 (2)
C9A—C1A—C10A—C11A	176.5 (2)	C12B—N2B—C11B—C10B	−55.1 (3)
C13A—C1A—C10A—C11A	52.8 (3)	C1B—C10B—C11B—N2B	55.4 (3)
C14A—N2A—C11A—C10A	−158.1 (2)	C14B—N2B—C12B—C13B	−151.31 (19)
C12A—N2A—C11A—C10A	56.5 (3)	C11B—N2B—C12B—C13B	54.7 (3)
C1A—C10A—C11A—N2A	−56.3 (3)	N2B—C12B—C13B—C1B	−53.9 (3)
C14A—N2A—C12A—C13A	157.7 (2)	O1B—C1B—C13B—C12B	−60.2 (2)
C11A—N2A—C12A—C13A	−54.9 (3)	C9B—C1B—C13B—C12B	178.10 (19)
N2A—C12A—C13A—C1A	52.8 (3)	C10B—C1B—C13B—C12B	54.1 (2)
O1A—C1A—C13A—C12A	61.0 (3)	C15B—N3B—C14B—O2B	−13.1 (8)
C9A—C1A—C13A—C12A	−176.4 (3)	C15C—N3B—C14B—O2B	1.9 (11)
C10A—C1A—C13A—C12A	−51.0 (3)	C15B—N3B—C14B—N2B	171.4 (8)
C15A—N3A—C14A—O2A	0.5 (4)	C15C—N3B—C14B—N2B	−173.7 (10)
C15A—N3A—C14A—N2A	177.4 (3)	C11B—N2B—C14B—O2B	173.9 (2)
C12A—N2A—C14A—O2A	−9.0 (4)	C12B—N2B—C14B—O2B	22.2 (3)
C11A—N2A—C14A—O2A	−153.3 (3)	C11B—N2B—C14B—N3B	−10.5 (3)
C12A—N2A—C14A—N3A	174.1 (2)	C12B—N2B—C14B—N3B	−162.2 (2)
C11A—N2A—C14A—N3A	29.8 (3)	C14B—N3B—C15B—C16B	162.7 (8)
C14A—N3A—C15A—C16A	−128.1 (4)	C15C—N3B—C15B—C16B	15 (3)
C2B—O1B—C1B—C9B	43.8 (3)	C14B—N3B—C15C—C16C	−106.6 (11)
C2B—O1B—C1B—C10B	164.34 (18)	C15B—N3B—C15C—C16C	−66 (4)
C2B—O1B—C1B—C13B	−78.8 (2)		

### Hydrogen-bond geometry (Å, º)

Cg1 is the centroid of the C4*B*–C9*B* benzene ring

**Table:**

*D*—H···*A*	*D*—H	H···*A*	*D*···*A*	*D*—H···*A*
N3*A*—H1*N*3···O2*A*^i^	0.81 (3)	2.12 (3)	2.915 (3)	170 (3)
N3*B*—H2*N*3···O2*B*^ii^	0.83 (2)	2.19 (3)	2.983 (3)	160 (2)
C11*A*—H11*B*···O2*A*^i^	1.00 (3)	2.27 (3)	3.246 (3)	165 (2)
C11*B*—H11*C*···O2*B*^ii^	0.95 (3)	2.50 (3)	3.344 (3)	147 (2)
C9*B*—H9*B*···O2*B*^iii^	0.92 (3)	2.58 (3)	3.473 (3)	163 (2)
C13*A*—H13*B*···*Cg*1^iv^	0.97	2.91	3.843 (3)	163

Symmetry codes: (i) *x*, −*y*+1/2, *z*−1/2; (ii) *x*, −*y*+3/2, *z*−1/2; (iii) −*x*+2, −*y*+1, −*z*+2; (iv) *x*, −*y*−1/2, *z*−3/2.
